# Two Outbreaks of Legionnaires Disease Associated with Outdoor Hot Tubs for Private Use — Two Cruise Ships, November 2022–July 2024

**DOI:** 10.15585/mmwr.mm7342a3

**Published:** 2024-10-24

**Authors:** Sooji Lee, Chris Edens, Troy Ritter, Luis O. Rodriguez, Kara Tardivel, Natalia A. Kozak-Muiznieks, Melisa Willby, Nancy Ortiz, Adam L. Cohen, Jessica C. Smith

**Affiliations:** ^1^Division of Bacterial Diseases, National Center for Immunization and Respiratory Diseases, CDC; ^2^Division of Environmental Health Science and Practice, National Center for Environmental Health, CDC; ^3^Division of Global Migration and Health, National Center for Emerging and Zoonotic Infectious Diseases, CDC.

**Summary**

**What is already known about this topic?**

Legionnaires disease is a serious pneumonia caused by *Legionella* bacteria. Hot tubs can be a source of *Legionella* growth and transmission when they are inadequately maintained and operated.

**What is added by this report?**

Epidemiologic, environmental, and laboratory evidence suggests that private balcony hot tubs were the likely source of exposure in two outbreaks of Legionnaires disease among cruise ship passengers. These devices are subject to less stringent operating requirements than are public hot tubs, and operating protocols were insufficient to prevent *Legionella* growth.

**What are the implications for public health practice?**

It is important for cruise ship operators to inventory hot tub–style devices across their fleets, evaluate the design features that increase the risk for *Legionella* growth and transmission, and test for *Legionella*.

## Abstract

Legionnaires disease is a serious pneumonia caused by *Legionella *bacteria. During November 2022–June 2024, CDC was notified of 12 cases of Legionnaires disease among travelers on two cruise ships; eight on cruise ship A and four on cruise ship B. CDC, in collaboration with the cruise lines, initiated investigations to ascertain the potential sources of on-board exposure after notification of the second potentially associated case for each ship. Epidemiologic data collected from patient interviews and environmental assessment and sampling results identified private hot tubs on selected cabin balconies as the most likely exposure source. To minimize *Legionella* growth, both cruise lines modified the operation and maintenance of these devices by removing the heating elements, draining water between uses, and increasing the frequency of hyperchlorination and cleaning. Hot tubs offer favorable conditions for *Legionella* growth and transmission when maintained and operated inadequately, regardless of location. Private hot tubs on cruise ships are not subject to the same maintenance requirements as are public hot tubs in common areas. Given the range of hot tub–type devices offered as amenities across the cruise industry, to reduce risk for *Legionella *growth and transmission, it is important for cruise ship water management program staff members to inventory and assess private balcony hot tubs and adapt public hot tub maintenance and operations protocols for use on private outdoor hot tubs.

## Investigation and Results

### Cruise Ship A Outbreak (November 2022–April 2024)

**FIGURE 1:**
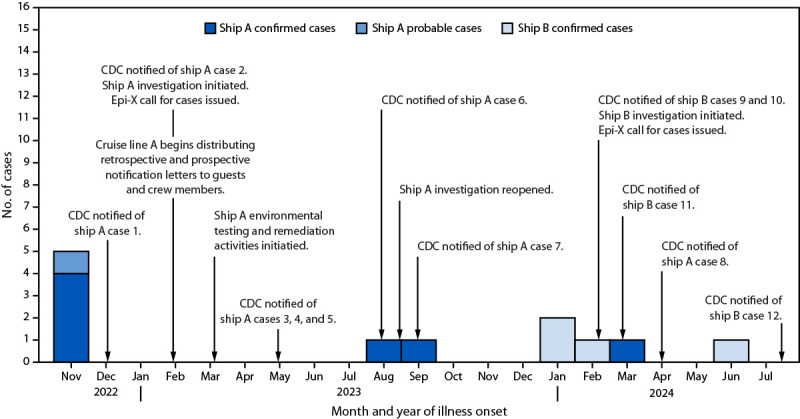
Investigation of two Legionnaires disease outbreaks* associated with private balcony hot tubs — two cruise ships, November 2022–July 2024 **Abbreviation:** Epi-X = Epidemic Information Exchange. * A confirmed Legionnaires disease case is defined as a clinically compatible case with confirmatory laboratory evidence for *Legionella*. A probable case is a clinically compatible case with an epidemiologic link during the exposure period without laboratory evidence for *Legionella*. https://www.cdc.gov/investigate-legionella/php/data-research/case-definitions.html

**FIGURE 2:**
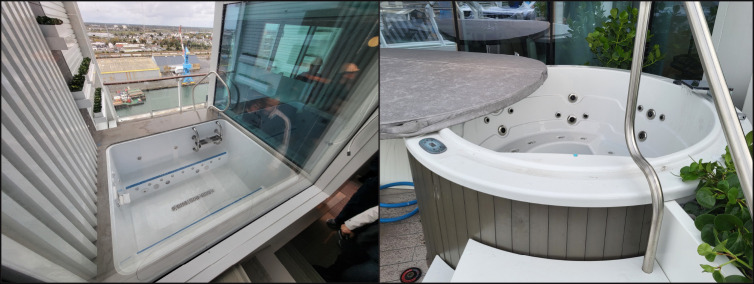
Images of hot tubs associated with cases of Legionnaires disease located on balconies only accessible via private cruise ship cabins* — two cruise ships, November 2022–July 2024 Photos/CDC Vessel Sanitation Program * Images are of devices before modification. Each device had components that can increase the risk for *Legionella* growth and transmission, including aerosol-generating jets, retention of water between uses, and presence of a heating element and recirculation and filtration systems.

During December 2022–May 2023, CDC was notified of five Legionnaires disease (LD) cases among patients (patients 1–5) who had traveled on cruise ship A during the 14-day exposure period* (Table) (Figure 1). All five cases (four laboratory-confirmed and one probable) were among passengers traveling on the same voyage in November 2022 (Supplementary Table; https://stacks.cdc.gov/view/cdc/165771) (*1*). During August–September 2023, two additional laboratory-confirmed cases with travel on different cruise ship A voyages were reported to CDC (patients 6 and 7). In April 2024, an additional laboratory-confirmed case was identified in a guest who traveled on cruise ship A the previous month (patient 8). No lower respiratory specimens were available; six patients were hospitalized, and no patients died. Local health departments interviewed patients to identify potential exposures on and off the ship, including hotel stays, health care visits, or other activities (Table). Patients 6 and 7 reported staying in cabins with a hot tub located on the private balcony (Figure 2).

**TABLE:** Selected characteristics of patients with confirmed and probable* Legionnaires disease — two cruise ships, November 2022–July 2024

Characteristic	No. (%) of Legionnaires disease cases	
	Cruise ship A n = 8	Cruise ship B n = 4
**Age, yrs**		
Age, mean (range)	70 (39–78)	63 (60–66)
Age, median	73	63
**Male sex**	6 (75)	3 (75)
**U.S. resident**	8 (100)	2 (50)
**Outcome**		
Hospitalized	6 (75)	4 (100)
Survived	8 (100)	4 (100)
**Disease status**		
Confirmed	7 (88)	4 (100)
Probable	1 (12)	0 (—)
**Potential exposures on board cruise ship**		
**Nights spent on board**		
No. of nights, mean (range)	8 (7–10)	9 (7–14)
No. of nights, median	7	8
**Potential exposure location**		
Spent time in or near public hot tubs	5 (63)	2 (50)
Stayed in cabin with private balcony hot tub	2 (25)	0 (—)
Visited the spa	5 (63)	0 (—)
Shoreside excursions during cruise voyage	4 (50)	2 (50)
Other potential travel exposures	3 (38)	2 (50)

* A confirmed Legionnaires disease case is defined as a clinically compatible case with confirmatory laboratory evidence for *Legionella*. A probable case is a clinically compatible case with an epidemiologic link during the exposure period without laboratory evidence for *Legionella*. https://www.cdc.gov/investigate-legionella/php/data-research/case-definitions.html

In response to notification of the second case in February 2023, CDC reviewed the vessel’s *Legionella* environmental sampling results from the preceding 6 months and water management program records. A total of 150 water samples were tested for *Legionella* during August 2022–February 2023 as part of the cruise line’s routine water management program validation. A single non-*pneumophila Legionella* detection was identified in the potable water system during that time (August 2022); after localized hyperchlorination of the water system, *Legionella* was not detected. All potable water parameters were within control limits and monitored according to CDC requirements^†^ (*2*). Review of operation and maintenance records for public hot tubs in common areas indicated that CDC requirements had been met (*2*). In March 2023, in response to the outbreak, the cruise line collected 260 1-L water samples from representative points of use, cabins of infected patients, heat exchangers, potable water tanks, decorative fountains, and public hot tubs in common areas. No *Legionella* was detected. The cruise line also conducted ship-wide hyperchlorination after sampling. An additional 76 potable and recreational water samples were collected during spring and summer 2023; no *Legionella* was detected.

In August 2023, upon identification of the case in patient 6, in which private balcony hot tub use was first reported, CDC requested all 10 private balcony hot tubs on the ship be closed and sampled because they had not been tested previously. *L. pneumophila* serogroup 2–14 (Lp2–14) and non-*pneumophila Legionella* species were detected in six of 10 hot tubs. Of the six private balcony hot tubs with *Legionella* detections, four had concentrations of *Legionella* >100 colony-forming units (CFU)/mL, and two had concentrations >1,000 CFU/mL. The hot tubs remained closed until their operation and maintenance protocols were modified and nondetectable *Legionella* sampling results were obtained. *Legionella* was not detected in environmental sampling of the potable water system or any recreational water features, including the balcony hot tubs, after the change in operation and maintenance protocols. During March 2024, when patient 8 traveled on ship A, to August 2024, approximately 300 samples were collected, and no *Legionella* was detected.

### Cruise Ship B Outbreak (January–June 2024)

During February–July 2024, CDC was notified of four confirmed LD cases in patients who traveled on cruise ship B during their exposure period (patients 9–12) (Table) (Figure 1). Two of the cases occurred in passengers traveling on the same voyage in January 2024 (patients 9 and 10); one of the passengers traveled on two consecutive voyages. The voyages of patients 11 and 12 were in February and May, respectively. Three patients received a positive *Legionella* urinary antigen test result, and one received a positive culture test result in which *L. pneumophila* was detected; four patients were hospitalized, and no patients died.

In response to the outbreak, CDC requested immediate closure of all hot tubs on the ship, including those in common areas and private balconies, and sampling of all hot tubs and representative potable water locations. *L. pneumophila* serogroup 1 (Lp1) and Lp2–14 species were detected in all eight private balcony hot tubs on the ship, and Lp2–14 was detected in a single location in the potable water system. Of the testing performed on the eight private balcony hot tubs, two samples had Lp1 concentrations >10 CFU/mL. All balcony hot tubs remained closed until each had nondetectable *Legionella* postremediation sampling results. As the cruise line implemented changes to the operation and maintenance of the balcony hot tubs, Lp1 and Lp2–14 continued to be detected in two of the eight hot tubs, prompting additional remediation efforts and further refinement of operational and maintenance protocols. This activity was reviewed by CDC, deemed not research, and was conducted consistent with applicable federal law and CDC policy.^§^

## Public Health Response

CDC published two Epidemic Information Exchange (Epi-X) calls for cases and notified the European Centre for Disease Prevention and Control to identify other cruise-associated patients with LD because both ships included itineraries in Europe. Cruise operators of both ships notified guests and crew of the potential for *Legionella* exposure while the investigations were ongoing. CDC reviewed illness logs from both ship clinics. CDC also notified cruise operators of the risk for *Legionella* growth associated with private balcony hot tubs during regularly scheduled calls with industry partners in December 2023 and June 2024.

Both cruise lines ultimately modified the operation and maintenance of the private hot tubs so that heating elements were removed; tubs were only filled upon guest request, drained between uses, and cleaned and disinfected more frequently. Ship A devices were additionally modified to remove filtration elements. Sampling is ongoing for both vessels.

## Discussion

Travel on cruise ships is a recognized risk factor for LD (*3*). CDC defines a cruise-associated outbreak as the occurrence of two cases in patients who had traveled on the same ship with voyages within 1 year of each other (*4*). In these investigations, both outbreaks involved patients with overlapping voyages, most notably ship A with five patients who traveled on the November 2022 voyage. The outbreak on cruise ship A is the largest cruise-associated LD outbreak investigated by CDC since 2008.

On ship A, the private balcony hot tubs were identified as a potential source of exposure after interviews with patients 6 and 7. These devices were found to be operating for months in a manner conducive to *Legionella* growth, which included maintaining a water temperature in the *Legionella* growth range (77°F–113°F [25°C–45°C]) for multiple days without draining and operating with no residual disinfectant. In addition, some of these devices were located on decks only one floor above or below common outdoor amenities; previous investigations have shown that hot tubs located in private areas can disseminate aerosols to common areas and result in exposures, even in persons who do not use the hot tubs themselves (*5*,*6*). Environmental testing revealed extensive *Legionella* colonization. Subsequent identification of *Legionella* in private balcony hot tubs operating on ship B strengthened the case that these devices were the likely exposure source.

According to current CDC requirements, private hot tubs are not required to have automated continuous disinfectant dosing and monitoring or pH monitoring, as is standard for public hot tubs. To meet CDC requirements, private hot tubs must only be shock-chlorinated, drained, and refilled weekly or between occupancies, whichever is sooner (*3*). Although the cruise lines adhered to current CDC requirements for operating and maintaining private hot tubs on ships A and B, these measures were insufficient to prevent *Legionella* growth.

### Limitations

The findings in this report are subject to at least three limitations. First, clinical isolates were not available for comparisons to determine genetic relatedness. Second, although clinical tests indicated patients were infected with Lp1, environmental testing detected other *Legionella* species and serogroups in the balcony hot tubs of ship A. However, the presence of any *Legionella* species indicates that conditions supporting growth existed in these devices. Finally, multiple patients reported other possible exposure locations during their travel, such as hotels and shoreside excursions at ports of call, although the cruise ships were the only common exposure among the infected patients.

### Implications for Public Health Practice

This report describes a previously unidentified source of *Legionella* exposure on cruise ships: hot tubs located on private cabin balconies, which have become more common as new ships enter service and older ones are renovated. A wide range of hot tub–style devices are used by cruise, hotel, and recreational water industries, including public hot tubs, jetted bathtubs, and hydrotherapy pools. Cruise lines and the hospitality industry should be aware of hot tub features that increase the risk for *Legionella* growth and transmission, including outdoor use, retention of water between uses, and the presence of recirculation, filtration, or heating systems.

Private outdoor hot tubs, as described in this report, are not unique to cruise ships A and B. Inventory of hot tub–style devices by cruise ship operators to ensure that they are included in the vessel’s water management program and are routinely tested for the presence of *Legionella* could help prevent cruise ship outbreaks of LD. Adapting public hot tub maintenance and operations protocols for use on private outdoor hot tubs can reduce the risk for *Legionella* growth and transmission.

## Footnotes

* A confirmed LD case is defined as a clinically compatible case with confirmatory laboratory evidence for *Legionella*. A probable case is a clinically compatible case with an epidemiologic link during the exposure period without laboratory evidence for *Legionella*. The likely exposure period is defined as the 14 days preceding symptom onset. https://www.cdc.gov/investigate-legionella/php/data-research/case-definitions.html

† The CDC Vessel Sanitation Program 2018 Operations Manual addresses current public health issues related to cruise ship sanitation. Requirements concerning the potable water disinfectant level and monitoring point are in Sections 5.4.1.2.1 and 5.5.1.1.1. Requirements related to public and private whirlpool spa operations are in Sections 6.3, 6.4, and 6.6. Public whirlpool spa requirements include continuous and automatic disinfectant dosing and monitoring, frequent shock halogenation, and filter cleaning and replacement procedures. “Whirlpool spa” is a synonymous term for “hot tub.” https://www.cdc.gov/vessel-sanitation/media/files/vsp_operations_manual_2018-508.pdf

§ 45 C.F.R. part 46, 21 C.F.R. part 56; 42 U.S.C. Sect. 241(d); 5 U.S.C. Sect. 552a; 44 U.S.C. Sect. 3501 et seq.
